# Waste By-Products in Asphalt Concrete Pavement Construction: A Review

**DOI:** 10.3390/ma18174092

**Published:** 2025-09-01

**Authors:** Nuha S. Mashaan, Daniel O. Oguntayo, Chathurika Dassanayake

**Affiliations:** Mineral Recovery Research Centre (MRRC), School of Engineering, Edith Cowan University, Joondalup, WA 6027, Australia; d.oguntayo@ecu.edu.au (D.O.O.); chatdasau@gmail.com (C.D.)

**Keywords:** bitumen modification, bitumen, asphalt mixture, mining waste, alternative aggregates and fillers, environmental impact, circular economy

## Abstract

The use of mining by-products in bitumen and asphalt mixture modification has drawn a lot of interest lately since it can improve pavement performance while advancing the goals of the circular economy and environmental sustainability. Mining by-products such as steel slag, red mud, silica fume, and fly ash have demonstrated good results as sustainable materials for improving the chemical, mechanical, durability, and rheological properties of asphalt binders and mixtures while also reducing the environmental degradation brought about by the disposal of these by-products. This study reviews research efforts on mining by-products (specifically steel slag, silica fume, red mud, and fly ash) in asphalt concrete pavement construction, analyzing the existing research, with emphasis on their various applications in asphalt concrete, their benefits as sustainable asphalt concrete materials, and limitations connected to their use. This review concludes by providing future directions in the utilization of these mining by-products in asphalt concrete production. This review contributes to the development of cost-effective, eco-friendly, and high-performance road construction materials, helping the transition to sustainable infrastructure.

## 1. Introduction

The rapid pace of urbanization, industrialization, and global infrastructure development has led to a continuously rising demand for construction materials, particularly within the transportation sector. Among these, asphalt mixtures are extensively utilized in the construction and rehabilitation of road pavements due to their favorable performance characteristics, cost-effectiveness, and ease of maintenance. However, the heavy reliance on non-renewable natural aggregates and bituminous binders poses significant environmental and economic challenges [1]. The extraction and processing of virgin materials are energy-intensive, contribute substantially to greenhouse gas emissions, and accelerate the depletion of finite natural resources. Consequently, there is increasing global concern about the sustainability of current pavement practices and an urgent need to investigate alternative materials that can mitigate the environmental impact of road construction [2,3].

One promising approach is the utilization of industrial and mining waste materials as substitutes for conventional fillers and aggregates in asphalt mixtures. The mining industry, particularly in resource-rich regions like Australia, produces enormous volumes of waste each year in the form of tailings, slag (wastes from metal production), fly ash, red mud, and other by-products. If improperly managed, these materials can pose significant environmental hazards, including land degradation, water pollution, and long-term ecological risks [4]. However, many of these wastes possess desirable physical and chemical properties that make them suitable for incorporation into pavement materials. When processed appropriately, these by-products can contribute to the structural integrity and durability of asphalt mixtures while simultaneously mitigating the environmental burden associated with their disposal [2,5].

The concept of sustainable road construction through the reuse of industrial by-products aligns with circular economy principles, which emphasize resource efficiency, waste minimization, and the creation of closed material loops. Rather than viewing mining waste solely as an environmental liability, this approach recognizes its potential as a valuable resource that can reduce the demand for virgin materials and enhance the performance of infrastructure systems. Moreover, integrating waste into road materials has economic benefits, such as reducing raw material costs, lowering transportation and disposal expenses, and supporting local industries by creating secondary markets for industrial residues [6,7]. Mining by-products, in particular, silica fume, slag, red mud, and fly ash, have a wide range of applications in asphalt concrete due to their availability and unique properties. Steel slag, a by-product of the steel-making process, is among the most studied materials for asphalt concrete applications. Due to its high density, angularity, and resistance to wear and polishing, steel slag serves as an effective coarse and fine aggregate. Studies have demonstrated that its inclusion in asphalt mixtures enhances properties such as rutting resistance, Marshall stability, and moisture susceptibility. Furthermore, steel slag’s inherent alkalinity contributes to improved binder adhesion and long-term durability [8,9]. Similarly, fly ash, derived from coal combustion in power plants, has been widely investigated as a filler material. Its pozzolanic activity, fine particle size, and spherical shape enable better packing and mastic stiffening, which, in turn, improves fatigue resistance and reduces oxidative aging of the bitumen [10,11].

Silica fume, another industrial by-product generated during the production of silicon alloys, exhibits an ultrafine particle size and high surface reactivity. These characteristics allow it to function as an effective binder extender and filler, promoting better dispersion, reducing air voids, and increasing adhesion between binders and aggregates. When incorporated in appropriate proportions, silica fume can significantly enhance mechanical performance metrics, including resistance to rutting, cracking, and fatigue loading [2,12]. On the other hand, red mud—an alkaline waste produced during bauxite refining—is increasingly being explored for asphalt modification. Though traditionally associated with environmental challenges due to its caustic nature, red mud offers unique benefits when stabilized and processed. It can improve high-temperature performance, reduce rutting depth, and contribute to UV and oxidation resistance in pavement applications [3,13].

While the mechanical advantages of mining waste incorporation are well-documented, the environmental benefits are equally compelling. Diverting waste from landfills or containment ponds and repurposing it into asphalt mixtures supports pollution control, conserves landfill space, and reduces greenhouse gas emissions associated with material production. Life cycle assessment (LCA) studies further reinforce the viability of this approach by quantifying the environmental savings achieved through partial or complete substitution of traditional asphalt constituents with mining waste materials. In some cases, incorporating industrial waste has led to more than 20% reductions in global warming potential, primarily through decreased energy consumption and material processing [2,3]. However, despite the amount of information supporting the utilization of waste by-products in asphalt concrete, this area of research could be regarded as ongoing, especially with mining wastes, specifically steel slag, red mud, fly ash, and silica fume, and their treatment for enhanced performance. This paper, therefore, conducts a comprehensive review of the waste by-products in asphalt concrete pavement construction, analyzing the existing research, with emphasis on their various applications and limitations connected to their use. This review concludes by providing future directions in the utilization of mining wastes in asphalt concrete production.

## 2. Incorporation of Mining Wastes as Bitumen Modifiers

The use of mining waste in bitumen modification has drawn a lot of interest lately since it can improve pavement performance while advancing the goals of the circular economy and environmental sustainability. This strategy tackles the demand for more resilient and environmentally friendly road materials as well as the efficient disposal of large mining by-products [13,14]. Mining wastes, specifically, steel slag, fly ash, silica fume, and red mud, possess unique chemical, mineralogical, and microstructural characteristics that can be harnessed to improve the mechanical, rheological, and thermal behavior of asphalt binders and mixtures. These materials typically exhibit pozzolanic activity, high surface area, and fine particle size, which allows them to interact efficiently with bitumen matrices and aggregate structures [12,15]. For instance, steel slag has been shown to significantly enhance the rutting resistance and stiffness of asphalt binders, primarily due to its angular particle shape and high calcium oxide content, which improve interlocking and load-bearing capacity [16]. Similarly, fly ash, a by-product of coal combustion, is widely recognized for its ability to improve the workability and fatigue resistance of asphalt mixtures, particularly in cold-mix asphalt applications. Its fine particle size and pozzolanic properties contribute to a denser and more cohesive mixture, resulting in enhanced long-term performance [17,18]. Silica fume, characterized by its ultrafine, nanoscale particles, is especially effective in increasing binder viscosity and improving resistance to moisture damage. Its high surface area facilitates better binder adhesion and reduces the potential for stripping under wet conditions [19]. In addition, red mud, an alkaline industrial residue from aluminum production, has demonstrated potential for improving the stiffness and durability of asphalt mixtures. However, its application requires cautious handling due to concerns related to moisture sensitivity and the leaching of heavy metals, which must be mitigated through appropriate stabilization and encapsulation techniques [10,20].

### 2.1. Bitumen

Bitumen binders are among the most widely used materials in construction and infrastructure applications due to their excellent adhesive and waterproofing capabilities. These binders are primarily used in road construction, waterproofing systems, roofing, and adhesives. Bitumen’s performance depends significantly on its chemical composition and physical behavior at varying temperatures and loading conditions [21]. The primary chemical components of bitumen are asphaltenes and maltenes, which include saturates (aliphatic hydrocarbons), aromatics (aromatic hydrocarbons), and resins (polar molecules, acting as a dispersant for asphaltenes). The heaviest and most intricate molecular constituents are asphaltenes, which increase viscosity and give structural rigidity. As adhesives, resins help asphalt mixtures stay cohesive and interact with particles. While saturates, being the least polar, contribute to bitumen’s fluidity and workability during mixing and compaction, aromatics operate as the oily medium that facilitates the dispersion of resins and asphaltenes [21].

### 2.2. Steel Slag

Steel slag, a by-product of the steel manufacturing process, has been widely studied for use in asphalt as both a filler and aggregate. Its high content of calcium, silica, and iron enhances interlocking, binder adhesion, and load distribution. The rough, angular, and microporous texture of steel slag promotes strong mechanical interlock and moisture resistance. Studies have shown that replacing traditional fillers with steel slag powder improves high-temperature deformation resistance, viscosity, and dynamic modulus [16,22]. Additionally, steel slag absorbs lighter asphalt constituents, enhancing mastic viscosity and promoting thermal stability. Electric arc furnace (EAF) steel slag containing mixtures exhibit improved tensile strength and resistance to rutting [23,24].

Furthermore, the presence of free lime (CaO) in untreated steel slag requires pre-treatment or aging to avoid volumetric instability. Studies by [8] have also shown improved Marshall stability, reduced voids in mineral aggregate (VMA), and enhanced fatigue life when using properly treated steel slag. The strong alkaline nature of steel slag improves adhesion with bitumen and reduces moisture susceptibility, especially in humid or wet conditions. XRF analysis confirms high CaO and Fe_2_O_3_ content, indicating pozzolanic reactivity and compatibility with acidic aggregates.

### 2.3. Fly Ash

Fly ash is a fine particulate by-product from coal combustion, rich in aluminosilicates. Its similar size and density to traditional fillers make it a suitable substitute. Fly ash improves Marshall stability and reduces the optimal bitumen content due to its stiffening effect and better particle packing [25]. The spheroidal and sometimes porous morphology of fly ash particles improves mix workability and binder absorption. It increases binder viscosity and reduces drain-down. Fatigue life is significantly improved [17], though moisture susceptibility remains neutral without treatment. In cold-mix applications, it enhances stability and durability [17,26]. Additionally, it has been demonstrated that fly ash enhances the mechanical and aging qualities of red mud.

Furthermore, figuring out how fly ash behaves in bituminous systems depends on whether it is classified as Class F (low-calcium) or Class C (high-calcium). While Class C fly ash might add extra binding qualities, Class F fly ash is usually more pozzolanic. SEM studies of fly ash containing asphalt reveal a denser microstructure and fewer air spaces, which enhances its long-term durability [11]. FTIR studies further confirm chemical bonding between fly ash particles and asphalt binders, particularly via hydroxyl and silanol groups.

### 2.4. Silica Fume

Silica fume is an ultrafine pozzolanic material from silicon and ferrosilicon alloy production, composed primarily of amorphous SiO_2_. Due to its nanoscale particle size and high surface area, silica fume effectively modifies asphalt rheology. Small additions (4–8% by binder weight) increase viscosity and the softening point, improving resistance to rutting and thermal degradation [27]. Silica fume also enhances oxidative aging resistance and moisture resistance [19]. Microstructural studies show that silica fume contributes to a well-dispersed, uniform binder matrix, increasing stress distribution, cohesion, and high-temperature performance.

Strong adsorption to bitumen is made possible by its high surface energy, which strengthens filler–binder linkages and thickens the structural bitumen film. According to [28], silica fume modified asphalt exhibits improved fatigue resistance and reduced phase angle (δ), indicating more elastic behavior. Additionally, its compatibility with other modifiers, like SBS or LDPE, enables hybrid modification strategies. SEM images display spherical particles ranging from 0.1 to 1 μm, forming a spatial network that contributes to thermal stability and durability.

### 2.5. Red Mud

Red mud is a by-product of alumina extraction from bauxite ore and contains high levels of iron oxide, aluminum oxide, and reactive alkaline compounds. Its fine particle size and alkalinity improve binder stiffness, adhesion, and anti-stripping properties. SEM analysis shows a denser, more cohesive binder matrix in red mud modified mixes [5]. Red mud enhances the softening point and viscosity while reducing penetration value, indicating better high-temperature stability [10]. Red mud also enhances moisture sensitivity and fatigue resistance when combined with fly ash or hydrated lime. However, stabilizing chemicals are frequently needed for its application because of its porosity.

Chemical analysis through XRF indicates a rich presence of Fe_2_O_3_ (28.4%), Al_2_O_3_ (17.5%), and SiO_2_ (12.6%), supporting its chemical reactivity in bituminous systems [20]. Strong molecular bonds with the asphalt binder and enhanced oxidative resistance are further suggested by FTIR measurements. Even though red mud has a lot to offer in terms of performance, encapsulation techniques or combined use with safer fillers should be used to address long-term environmental concerns, including possible heavy metal leaching [29]. Table 1 presents the comparative oxide composition of three major mining wastes—red mud, steel slag, and fly ash—commonly used in the modification of bituminous materials. The presence and proportion of oxides such as Fe_2_O_3_ (iron oxide), Al_2_O_3_ (aluminum oxide), SiO_2_ (silicon oxide), CaO (calcium oxide), and alkali oxides (Na_2_O + K_2_O) are key indicators of their reactivity and compatibility with bitumen.

Table 2 presents a summary of key observations of different chemicals in the mining wastes used in bitumen modification.

### 2.6. Physical Properties

The physical properties of bitumen-modified mining wastes are fundamental indicators of pavement performance, such as their penetration value, softening point, and viscosity. Mainly, performance is judged in terms of workability, temperature susceptibility, and resistance to deformation.

#### 2.6.1. Penetration Value

**Figure 1:**
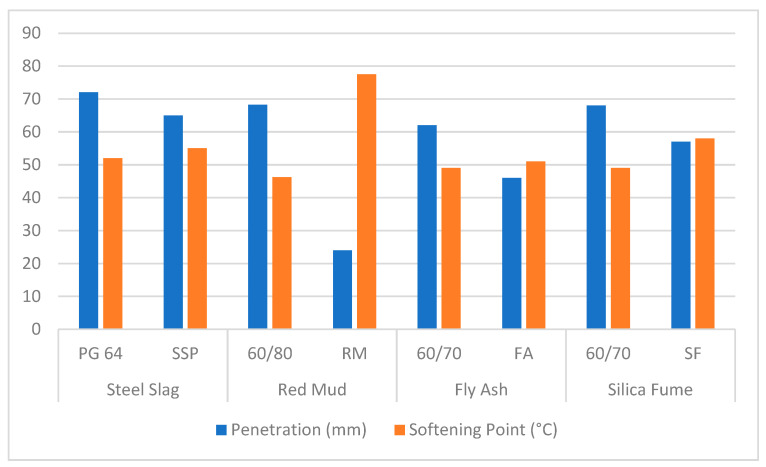
Penetration and softening point of mining waste-modified bitumen [32,33,34,35,36].

The penetration value of bitumen is a standard measure of its consistency, reflecting how easily the material deforms under specific loading and temperature conditions. It is determined by the depth (in tenths of a millimeter) that a standard needle penetrates a bitumen sample under a load of 100 g applied for 5 s at 25 °C. This parameter is particularly significant in evaluating the stiffness of a binder and its suitability under varying climatic conditions. As depicted in Figure 1, incorporating industrial by-products, such as steel slag, fly ash, and red mud, typically results in a decrease in penetration values, indicating a harder and stiffer binder [30,31]. If not properly balanced, this increase in stiffness may decrease flexibility at low temperatures, but it is advantageous for rutting resistance, particularly in high-temperature areas. Table 2 presents a summary of key observations of different chemicals in mining wastes used in bitumen modification.

Penetration value assesses the consistency and hardness of bitumen by measuring the depth to which a standard needle penetrates a binder under a specific condition. Modified binders, mainly those that incorporate red mud, fly ash, and steel slag, have shown a decrease in the penetration values compared to traditional bitumen, which indicates an improvement in stiffness and resistance to deformation under load [10,12]. These findings highlight the potential of waste-derived additives not only in enhancing the mechanical performance of asphalt binders but also in promoting more sustainable pavement materials.

#### 2.6.2. Softening Point

The softening point provides an indication of the thermal susceptibility of bitumen, defined as the temperature at which the binder transitions from a semi-solid to a more fluid state. The Ring and Ball test is commonly employed to determine this property. Higher softening points are indicative of better performance under high-temperature conditions, as they suggest improved resistance to deformation and flow. Studies have demonstrated that the integration of materials such as electric arc furnace slag, copper slag, and other mineral-based industrial wastes increases the softening point of modified binders, attributable to their high melting points and stiffening effects [32]. Asphalt mixtures can better tolerate higher service temperatures when mining waste fillers are included because they raise the softening point (see Figure 1). According to studies, binders changed with red mud have softening points that are 8–15 °C higher than those of unmodified binders. This results in enhanced resistance to rutting [14,23].

#### 2.6.3. Viscosity

Viscosity represents the internal resistance of bitumen to flow and is essential in determining its workability, pumpability, and coating ability during mixing and compaction. It is generally measured using a Brookfield Rotational Viscometer at elevated temperatures (typically 135 °C and 165 °C). Modifying bitumen with mining wastes, such as fly ash or red mud, can influence viscosity in ways that either enhance or hinder performance, depending on the type, fineness, and dosage of the additive. Increased viscosity typically enhances the binder’s ability to maintain aggregate coating during service, thereby improving durability and moisture resistance [37].

Viscosity shows the flow resistance of the binder and its other characteristics. While modifiers such as fly ash help to increase viscosity due to enhanced binder–filler interactions, warm-mix additives and sulfur also show potential to decrease mixing and compaction temperatures without compromising performance. High viscosity at mixing temperatures mostly shows better binder stiffness and elastic recovery [15,38].

#### 2.6.4. Dynamic Shear Rheometer

A key component of the Superpave binder specification is the Dynamic Shear Rheometer (DSR) test, which measures the complex shear modulus (G*) and phase angle (δ) to assess the viscoelastic behavior of bituminous binders. These factors aid in forecasting the resistance of asphalt concrete to fatigue cracking and rutting under frequent traffic loads. Greater rutting resistance at high temperatures is correlated with a higher G*/sinδ ratio, whereas fatigue performance at intermediate temperatures is assessed using G*·sinδ. These rheological properties are improved by adding nano- and microparticles from mining waste because of better particle–matrix interaction and filler effects [39,40,41]. The effects of modified bitumen on *complex shear modulus (G) and phase angle (δ)**, which indicate resistance to deformation under loading, have been assessed in several experiments using the Dynamic Shear Rheometer (DSR).

#### 2.6.5. Aging

Bitumen undergoes aging during its lifecycle, which includes short-term aging during mixing and compaction and long-term aging during service. These processes result in oxidation, volatilization, and increased stiffness, leading to potential cracking and reduced service life. Standard simulation methods such as the Rolling Thin Film Oven Test (RTFOT) and Pressure Aging Vessel (PAV) are employed to assess aging susceptibility. Research indicates that mining waste materials with pozzolanic or antioxidant properties—such as red mud, fly ash, and certain slags—can mitigate the effects of oxidative aging by absorbing free radicals or creating a denser microstructure that limits oxygen penetration [42,43].

Table 3 below summarizes the findings of the physical properties of modified bitumen.

### 2.7. Mechanical Properties

Surface free energy analysis indicated improved adhesion and moisture resistance when alkaline wastes, like steel slag and red mud, were used, as demonstrated by multiple moisture susceptibility tests. Certain waste modifiers can help with fatigue and thermal cracking. It was discovered that adding geopolymer additives (produced from fly ash or metakaolin, which are mineral wastes) improved the resistance to cracking at low temperatures. For example, adding 12% fly ash geopolymer to a binder reduced its stiffness at low temperatures, raising the binder’s Performance Grade from −22 °C to −28 °C. However, if not utilized carefully, highly stiff waste (such as high dosages of plastics or other industrial slags) might increase the asphalt mixture’s susceptibility to fatigue. In conclusion, the proper incorporation of mining/industrial wastes can improve the high-temperature performance (rutting resistance) of asphalt binders and mixtures, according to rheological tests (DSR, phase angle) and mechanical tests (Marshall stability, dynamic modulus, creep, etc.) [14].

### 2.8. Chemical Properties

Long-term performance and interaction mechanisms are significantly influenced by the chemical properties of bitumen and its modifiers. To assess these characteristics in waste-modified binders, sophisticated analytical methods, like Fourier transform infrared spectroscopy (FTIR), attenuated total reflectance (ATR), differential scanning calorimetry (DSC), X-ray diffraction (XRD), and X-ray fluorescence (XRF), are frequently employed.

#### 2.8.1. Fourier Transform Infrared Spectroscopy (FTIR)

Fourier transform infrared spectroscopy (FTIR) is a powerful analytical technique used to identify functional groups in materials by measuring their infrared absorption spectra. This method provides detailed information about the molecular composition and structure of both organic and inorganic substances. In the context of mining waste characterization, FTIR plays a vital role in detecting functional groups, such as hydroxyl (–OH), carbonate (CO_3_^2^^−^), silicate (SiO_4_^4^^−^), and sulphate (SO_4_^2^^−^). The presence of these groups indicates the occurrence of minerals commonly found in mining residues, including gypsum, clay minerals, and quartz [44]. By identifying these chemical signatures, FTIR contributes to a deeper understanding of the mineralogical composition, which is essential for assessing a material’s potential reactivity and environmental impact. Specifically, it allows for the evaluation of potential pollutants, the pozzolanic activity (i.e., the ability to react with calcium hydroxide to form cementitious compounds), and the chemical compatibility of mining waste with traditional binders used in construction applications. This information is critical for determining the feasibility of incorporating such waste materials into sustainable construction practices [42,44,45].

#### 2.8.2. Attenuated Total Reflectance (ATR)

ATR is a surface-enhanced sampling technique commonly paired with FTIR. It allows direct analysis of solid and powdered mining waste with minimal sample preparation. ATR is particularly effective in identifying surface-level chemical bonds and alterations due to treatments like heat activation or chemical modification [45].

#### 2.8.3. Differential Scanning Calorimetry (DSC)

DSC evaluates thermal behavior by recording heat flow during heating or cooling. It is used to assess phase transitions such as dehydration, crystallization, or decomposition. In mining waste, DSC reveals changes in thermal stability and indicates whether certain components, like hydrated minerals or organic residues, may react at elevated temperatures [46]. The behavior of changed binders during heat transitions is revealed by DSC (differential scanning calorimetry). Research using LDPE, sulfur, and nano silica showed enhanced thermal stability and a change in the glass transition temperature (Tg). The enhanced elasticity and phase stability of changed binders at varying temperature ranges are supported by these findings [15].

#### 2.8.4. X-Ray Diffraction (XRD)

XRD provides mineralogical analysis by identifying crystalline phases based on diffraction patterns. It is instrumental in determining the mineral composition of mining waste, such as hematite, quartz, feldspar, calcite, or hydrogarnet, which influence its suitability for road applications. XRD also helps detect amorphous or poorly crystalline phases, critical for understanding long-term behavior [44]. Crystalline phases like hematite, gibbsite, and quartz were verified to be present in red mud and fly ash. Additionally, XRD studies confirmed the idea that adding red mud and silica fume to modified binders leads to increased crystallinity and physical stability [10,14].

XRD offers a rapid, non-destructive method for determining elemental composition, including oxides (e.g., SiO_2_, Al_2_O_3_, Fe_2_O_3_, CaO) and trace metals (e.g., Pb, Cr, As). These data are critical for evaluating the environmental impact and classification of waste as inert or hazardous. The chemical data from XRF support mix design and quality control in bitumen modification [44,45]. XRD analysis has been applied to determine the oxide compositions of red mud, fly ash, and steel slag. High concentrations of Fe_2_O_3_, Al_2_O_3_, SiO_2_, and CaO were consistently reported, reinforcing their potential chemical reactivity in bitumen matrices and compatibility with acidic aggregates [5,20]. Table 4 below shows that red mud is chemically rich in different chemicals that can contribute to asphalt binder modification, such as improving stiffness, promoting adhesion, and reducing stripping.

Table 5 below shows the key findings for each of the chemical properties of mining wastes used as bitumen modifiers. FTIR spectra confirm the presence of functional groups such as hydroxyl, carbonate, silicate, and sulphate, indicating mineral constituents like quartz, gypsum, and clay. These findings are critical for assessing pozzolanic activity, chemical stability, and compatibility with binders. Overall, the data summarized in Table 5 underscore the diverse performance potential of different waste materials, suggesting that their successful incorporation into construction applications depends on a tailored approach that considers their unique physical and chemical characteristics. These insights provide a strong foundation for optimizing mix designs and enhancing the sustainability of civil engineering practices.

### 2.9. Microstructural Properties

The morphology, dispersion quality, and interfacial interactions of asphalt binders treated with mining waste can all be thoroughly understood using microstructural analytical techniques. Modified asphalt binders have been extensively characterized at the micro- and nanoscale levels using methods like Atomic Force Microscopy (AFM), energy dispersive X-ray spectroscopy (EDX/EDAX), mapping (MAPP), Scanning Electron Microscopy (SEM), and Field Emission Scanning Electron Microscopy (FESEM) [47,48,49,50,51,52].

#### 2.9.1. Scanning Electron Microscopy

Scanning Electron Microscopy (SEM) provides detailed surface morphology of asphalt mixtures and fillers. This method is particularly useful for observing the dispersion, bonding, and porosity of the particles within the asphalt matrix. SEM analyses have shown that materials such as red mud, fly ash, and steel slag possess irregular particle structures with rough surfaces that can enhance mechanical interlock and bonding with the binder [48,52]. SEM analysis has been instrumental in observing the surface morphology of red mud, steel slag, and fly ash particles, as well as their dispersion within the bitumen matrix, and has demonstrated that red mud-modified asphalt mixtures exhibit a more cohesive and denser microstructure compared to conventional mixtures. Their SEM images reveal finer distributions and better interfacial bonding, which are essential for durability [20]. Macroscale performance gains are corroborated by these microscopic data; for example, improved adhesion and internal friction from abrasive waste particles enhance resistance to rutting and stripping [9].

#### 2.9.2. Field Emission Scanning Electron Microscopy

FESEM is a higher-resolution variant of SEM that offers ultrafine imaging, allowing precise visualization of nanoscale features. In studies involving nanoparticle additives or modified binders with mining waste, FESEM has been used to observe fine structural transitions and crack propagation resistance mechanisms [50,51]. The FESEM technique has provided further confirmation of element dispersion and filler–matrix compatibility. Elemental mapping shows that oxides such as Fe, Al, and Si from red mud and slag are uniformly embedded within bitumen, affirming strong physicochemical integration [20].

#### 2.9.3. Mapping and Phase Profiling

Mapping and phase profiling involve combining SEM imaging with energy dispersive spectroscopy (EDS) or X-ray techniques to spatially analyze different material phases. This method has been employed to visualize the distribution of mineral phases such as hematite, alumina, and silica in red mud and fly ash within asphalt matrices [53,54]. In addition, MAPP analysis has supported visual confirmation of the uniform spread of particles in composite binders, particularly when combining low-density polyethylene (LDPE) or sulfur with steel slag, and has noted smoother surfaces and fewer voids in modified samples, corresponding with improved compatibility and lower air voids [12].

#### 2.9.4. Energy Dispersive X-Ray Analysis

Energy dispersive X-ray spectroscopy (EDS or EDAX) is commonly used in combination with Scanning Electron Microscopy (SEM) to analyze the elemental composition of fillers and binders. This technique has proven valuable in confirming the presence of key elements such as calcium (Ca), silicon (Si), aluminum (Al), and iron (Fe) in materials like steel slag, fly ash, and red mud. These elements play a crucial role in enhancing the reactivity and mechanical performance of binders, contributing to improved strength and durability in construction applications [47].

#### 2.9.5. Atomic Force Microscopy

AFM provides high-resolution topographical imaging and allows for the measurement of surface roughness and mechanical properties at the nanoscale. It has been used to analyze the interface between binders and modified fillers, including adhesion and viscoelastic behavior. AFM studies have revealed how nanostructured modifiers improve fatigue resistance and moisture susceptibility in modified asphalt [49,55]. AFM studies demonstrated increased surface roughness and elasticity in silica fume modified binders, contributing to improved stress distribution and fatigue resistance [56].

Table 6 below shows a summary of findings for different microstructural properties.

## 3. Use of Mining Wastes as Fillers and Aggregates in Asphalt Mixtures

Aggregates and fillers hold significant significance in ensuring road pavements’ structural integrity and resilience against external pressures. They play a crucial role in asphalt concrete pavement by substantially impacting its longevity, ecological sustainability, and overall effectiveness; they constitute about 95% of the makeup of asphalt concrete. Therefore, reduced greenhouse gas emissions and the depletion of limited natural resources frequently used as aggregates and fillers are necessitated through the use of mining wastes. The utilization of mining wastes such as red mud, steel slag, fly ash, and other mining by-products as alternative fillers and aggregates in asphalt mixtures has gained considerable attention due to their economic and environmental benefits [2,4]. These materials, which were traditionally regarded as waste, offer comparable or even superior performance compared to conventional materials when processed and integrated correctly [3].

### 3.1. Steel Slag

Steel slag is a by-product of the steel-making process and has gained considerable attention as a substitute for conventional aggregates in asphalt mixtures. It is known for its high angularity, hardness, and resistance to polishing, making it particularly suitable for enhancing the skid resistance and stability of road surfaces [57]. It is commonly used as both a coarse aggregate and a mineral filler. Its angular texture and high calcium and iron content contribute to improved rutting resistance, Marshall stability, and moisture damage resistance [58]. Replacing natural aggregates with steel slag can result in a higher resilient modulus and tensile strength, while slag filler has been shown to enhance surface free energy and cohesion in micro-surfacing treatments [8].

It is also found that extensive application is a coarse and fine aggregate. According to studies, slags from basic oxygen furnaces (BOF) and electric arc furnaces (EAF) improve asphalt mixtures’ fatigue life, tensile strength, and stiffness modulus [23,24]. Because steel slag is alkaline, it also helps to improve moisture resistance when used as filler. It is hard, angular particles and microporous surface texture promote excellent asphalt–aggregate adhesion, which translates to enhanced rutting resistance and lower permanent deformation under load [59].

### 3.2. Fly Ash

Fly ash, a residue from coal combustion, is rich in aluminosilicate compounds and has shown promise as a filler in asphalt mixtures. It has been observed that fly ash improves the workability and stiffness of asphalt binders, contributing to reduced deformation under traffic loads. Moreover, its use supports waste valorization and provides an environmentally responsible alternative to traditional fillers [11]. It is a combustion by-product from coal-fired power plants, and its role as a filler has also been investigated. Research has demonstrated that fly ashes of Class F (siliceous) and Class C (high calcium) exhibit similar water resistance and Marshall stability to limestone fillers. The tiny, spherical particles of fly ash increase mastic stiffness and packing, which can occasionally improve high-temperature performance and lessen asphalt oxidation [11].

It is also often used as a filler due to its pozzolanic reactivity. It contributes to better coating of aggregates, improved binder interaction, and enhanced fatigue resistance. Its spherical shape and smooth texture help improve workability during mixing and compaction [10]. Additionally, fly ash’s spherical particles improve the workability of the asphalt mastic and may reduce the optimum binder content by aiding compaction [60]. On the downside, very high fly ash contents might slightly reduce moisture resistance (as fly ash is hydrophilic), but moderate usage (e.g., 5–10% of mix) has shown net benefits or at least neutral effects on moisture sensitivity [18,61].

### 3.3. Silica Fume

Silica fume’s small particle size and pozzolanic activity offer potential enhancements in fatigue and rutting resistance [2]. Additionally, ferrochrome slag, silica fume, and recovered asphalt pavement (RAP) have been incorporated into hot-mix and cold-mix asphalts. These aggregates and fillers show promise for enhancing asphalt pavements’ durability and mechanical performance. For instance, ferrochrome slag increases skid resistance and rigidity, whereas silica fume promotes adhesion and filler–binder interaction. When properly integrated, such materials fill micro-voids and improve cohesion between asphalt and aggregates [12,15]. Due to its extremely small particle size (~0.1 μm) and high silica content, silica fume can dramatically increase the density and cohesion of the asphalt mastic. Even a few percent of silica fumes in the binder can reduce the temperature susceptibility and improve the aging resistance of asphalt mixtures [62]. Recent work by Hou et al. [63] on bio-asphalt binders (bitumen blended with bio-oil) showed that silica fume modification significantly improved the binder’s rheological properties. Specifically, at 10% silica fume, the rutting factor increased by 591.8% and the rutting factor aging index (RFAI) decreased by 54.7%, indicating greater rutting resistance. It also mitigated oxidative aging effects.

### 3.4. Red Mud

Red mud, either naturally occurring or derived from mining and metallurgical processes, has been used in asphalt mixtures to improve thermal conductivity, aging resistance, and color modulation. Its inclusion can enhance the high-temperature performance of asphalt binders and reduce the urban heat island effect when used in lighter-colored pavements [59]. Moreover, red mud improves UV resistance and extends the service life of asphalt surfaces when used in modified binders or as a pigment additive [61]. Red mud enhances stability at high temperatures and resistance to irreversible deformation, according to studies. For example, compared to control mixtures, mixes containing 3–7% red mud have shown decreased rut depths and improved Marshall stability [3,7]. However, if red mud is not combined with additional stabilizing agents like hydrated lime, it can shorten fatigue life and slightly increase air voids and binder demand because of its porosity [5]. Additionally, anti-stripping chemicals can reduce moisture susceptibility, which may slightly increase but remain within acceptable bounds [64]. According to Zhang et al. [13] and Kumar and Ramakrishna [20], red mud not only meets filler criteria but also enhances asphalt mixture durability due to its high alumina and iron content. For example, a study that investigated a micro-surfacing mix with red mud filler observed notable 22.6% and 11.5% increases in moisture resistance and raveling resistance compared to a traditional filler, respectively [65].

Table 7 below summarizes the findings of mining waste as alternative fillers and aggregates in asphalt mixtures.

### 3.5. Mechanical Properties

The endurance of asphalt mixtures is largely determined by their mechanical performance, particularly when subjected to environmental conditions and frequent loading. Significant gains in rutting resistance, cracking resistance, fatigue performance, and moisture susceptibility have been shown when mining waste materials, including red mud, steel slag, fly ash, and silica fume, are used [7,8,11,12,57].

#### 3.5.1. Rutting Resistance

**Figure 2:**
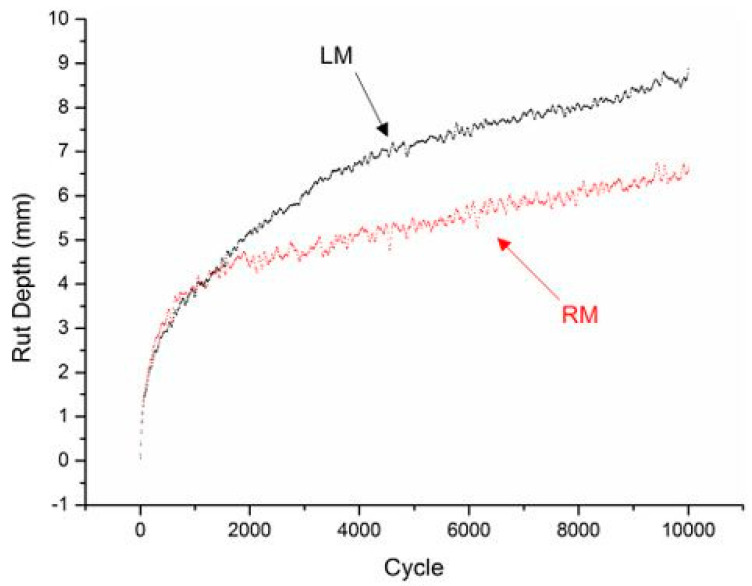
Rutting resistance of red mud-modified asphalt concrete (LM = Limestone, RM = Red Mud) [7].

One of the main distress modes in asphalt pavements is rutting, which is the permanent distortion of the wheel path brought on by high temperatures and strong traffic loads. It has been shown that adding mining wastes, like red mud and steel slag, to asphalt mixtures improves their resistance to rutting. Red mud increases stiffness at high temperatures, whereas steel slag’s high angularity and hardness improve load distribution and resistance to shear deformation [3,57]. As shown in Figure 2, asphalt mixes with 3–7% red mud showed a significant reduction in rut depth compared to control mixtures [7]. For instance, a study by Bhupathi et al. [66] demonstrated that incorporating steel slag into asphalt mixtures significantly enhanced the rutting resistance by 23.71%. This increase was attributed to its crystalline structure and high calcium and silicon content, which improved the stiffness and load-bearing capacity of the pavement. Rutting resistance is notably enhanced by incorporating angular and rough-textured materials like steel slag, which provide better interlock and load distribution. The authors of [58] and [64] reported that asphalt mixtures containing electric arc furnace (EAF) steel slag exhibited lower permanent deformation in wheel tracking tests, particularly at elevated temperatures. Red mud has also shown potential in resisting rutting due to its contribution to binder stiffness. By combining flexible modifiers, like sulfur or LDPE, with steel slag or red mud, cracking resistance is increased. Mehmood et al. [12] found that asphalt concrete samples amended with sulfur and polyethylene had less crack propagation, which they attributed to improved microstructure cohesion and binder elasticity.

Similarly, copper slag as a sand/filler replacement led to higher Marshall stability and rutting strength, especially when larger slag particle sizes were used, which contributed to greater mixture strength under moisture and temperature stress [67].

#### 3.5.2. Cracking Resistance

Cracking resistance, especially low-temperature and reflective cracking, is critical for maintaining pavement integrity. Silica fume and fly ash, due to their pozzolanic reactivity, contribute to binder modification and improved flexibility. These additives enhance the stress absorption capacity of asphalt mixtures, delaying the formation and propagation of cracks [11,12]. The spherical particles in fly ash improve workability, reduce voids, and lead to better compaction, which collectively support crack resistance [10].

Additionally, recent studies by Zheng et al. [28] indicated that incorporating silica fume into asphalt mixtures could significantly improve low-temperature cracking resistance. The study found that a 6% addition of silica fume enhanced flexibility and reduced the brittleness of an asphalt binder, thereby improving its performance in colder climates. Some waste modifiers, especially fine fillers like silica fume or fly ash, stiffen the binder significantly, which can reduce the mixture’s ability to flex under repeated loads. However, many studies report that fatigue performance remains acceptable or even improves when waste is used in moderation. For instance, mixtures incorporating steel slag, which is stiffer than limestone, have shown comparable or slightly better fatigue lives in controlled tests [68]. On the other hand, some fillers can enhance crack resistance by improving the binder’s healing ability; for example, mixtures with electric arc furnace dust filler have been reported to exhibit longer fatigue lives and self-healing capacity due to the dust’s chemical interaction with bitumen [69].

#### 3.5.3. Fatigue Resistance

Fatigue failure occurs due to the repeated loading of pavements over time. Materials like steel slag and silica fume have been linked to enhanced fatigue resistance. The dense matrix and improved binder–aggregate interaction created by these fillers absorb energy from cyclic loads and resist crack initiation [2,8]. The incorporation of EAF and BOF steel slags has been associated with longer fatigue life and improved tensile strength [23,24].

**Figure 3:**
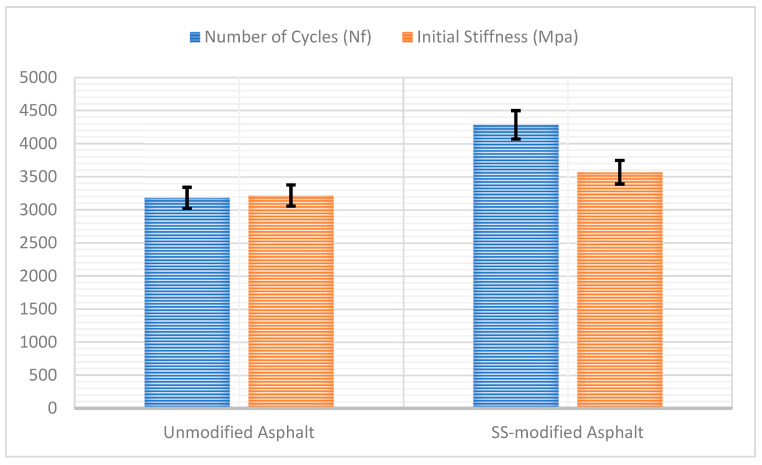
Fatigue resistance of steel slag modified asphalt concrete [10].

Further supporting this, a study by Zheng et al. [28] revealed that asphalt mixtures modified with silica fume exhibited superior fatigue resistance compared to conventional mixtures. The enhanced performance was attributed to the improved elasticity and reduced stiffness of the binder, which allowed it to better withstand repeated loading cycles. According to [10], steel slag (SS) improved the fatigue life of asphalt mixtures by 33.8% under cyclic loading circumstances and decreased the onset of cracks (as shown in Figure 3).

#### 3.5.4. Moisture Susceptibility

**Figure 4:**
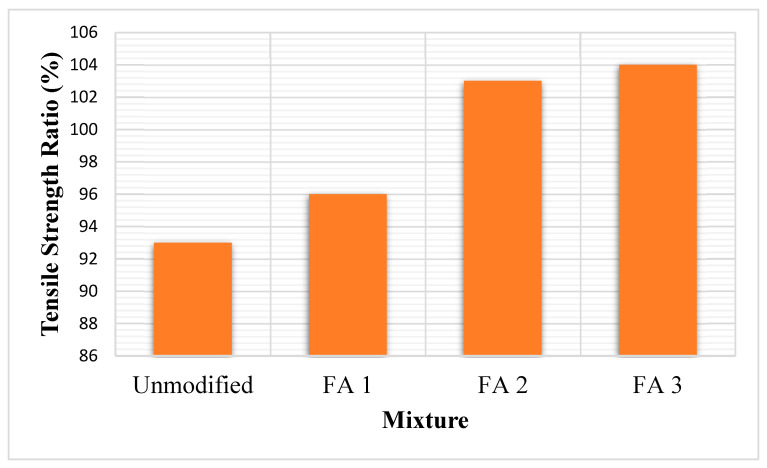
Moisture resistance of fly ash modified asphalt concrete [11].

Moisture susceptibility is a critical concern, particularly in wet environments, as it affects the adhesion between binders and aggregates. Steel slag and fly ash, due to their alkaline and pozzolanic properties, respectively, enhance moisture resistance by improving adhesion and reducing water intrusion. Steel slag’s alkalinity increases surface free energy, leading to better binder bonding [8]. As depicted in Figure 4, fly ash (FA) contributes to reduced moisture damage and improved film thickness over aggregates [11]. However, red mud, due to its porosity, may slightly increase moisture sensitivity unless treated with anti-stripping agents [6,13]. Recent studies by Bhupathi et al. [66] showed that incorporating steel slag into asphalt mixtures not only improves mechanical properties but also enhances moisture resistance. The studies attributed this improvement to the slag’s dense structure and chemical composition, which reduced water permeability and improved the durability of the pavement. Fly ash can also contribute to moisture resistance by filling micro-voids and reducing permeability. In a warm-mix study, replacing 4–8% of a filler with fly ash improved the mix’s tensile strength ratio (TSR), indicating better resistance to stripping [70,71].

Lastly, the addition of alkaline fillers, such as steel slag and red mud, greatly reduces moisture susceptibility, a typical cause of pavement failure. By strengthening the adhesive bond between aggregates and binders, these compounds reduce stripping and damage from water. Mixtures including these waste materials exhibit a percentage increase of 5–10% in properties like the TSR and Indirect Tensile Strength (ITS), indicating improved moisture resistance [14].

Table 8 below shows a summary of the findings for the mechanical properties of different mining wastes used as alternative fillers and aggregates in asphalt mixtures.

### 3.6. Environmental and Circular Economy

As a non-conventional material in asphalt concrete, waste by-products are a promising solution for both asphalt concrete and the environment, as they have been proven to improve the performance of asphalt concrete. There are some potential trade-offs to consider, even though the environmental advantages of using polymeric modifiers and mining waste fillers in asphalt mixtures are widely established. Heavy metals have been discovered to be present in several mining waste-based fillers, like red mud, which raises questions about their potential for long-term leaching [23]. Furthermore, even though steel slag has great mechanical qualities, its high free lime content may cause volumetric expansion, necessitating pre-treatment procedures prior to asphalt mixture application [14]. Beyond technical performance, the use of mining and industrial wastes in asphalt has significant environmental implications. Life cycle assessment (LCA) studies indicate that substituting virgin materials with these wastes can substantially reduce the overall environmental footprint of pavement construction [72]. Similarly, the incorporation of fly ash and red mud as fillers can decrease the energy and carbon footprint of asphalt concrete production by offsetting cement or lime usage and diverting those wastes from landfills [73,74].

To overcome these obstacles and guarantee that fillers made from industrial wastes continue to be both environmentally safe and structurally sound for long-term pavement applications, more research is required on stabilization methods, leachate control strategies, and field performance monitoring [10]. Life cycle assessment (LCA) studies indicate that the substitution of natural fillers with industrial wastes can significantly lower the environmental impact of asphalt concrete production. For instance, the use of red mud and fly ash has been reported to reduce embodied energy and global warming potential due to the avoidance of high-energy mineral filler production processes [24,75,76]. Similarly, replacing conventional aggregates with steel slag reduces quarrying-related impacts, including dust emissions, noise, and land degradation [23,77].

Several case studies of carbon footprint analyses attest to the fact that using mining waste reduces greenhouse gas emissions. CO_2_ equivalent emissions are reduced in asphalt mixtures that comprise waste-based geopolymers or recycled industrial by-products, especially during the hot-mix preparation and binder synthesis processes. When steel slag is used with Sasobit or LDPE in certain warm-mix applications, the necessary mixing temperatures are further decreased, which indirectly lowers fuel consumption and CO_2_ emissions [38]. Additionally, by turning vast amounts of by-products into useful materials for road construction, mining waste reuse supports the circular economy. For example, millions of tons of red mud are created each year, and leaching and disposal are major problems. Incorporating industrial waste materials into bituminous mixtures not only helps divert significant volumes of waste from landfills but also supports the principles of closed-loop material recovery and the circular economy. By reusing these by-products in pavement construction, the life cycle of valuable raw materials is extended, reducing the demand for virgin resources and minimizing environmental burdens. In particular, the reintegration of fly ash and silica fume, generated from thermal power plants and steel manufacturing processes, into asphalt concrete has demonstrated both functional and environmental benefits. These include enhanced mechanical properties, such as improved durability, stiffness, and resistance to moisture damage, as well as a reduced carbon footprint associated with pavement production and maintenance [15].

## 4. Conclusions

The increasing urgency of addressing environmental degradation, resource depletion, and industrial waste management has prompted the construction industry to seek sustainable alternatives to conventional materials. This paper presents a comprehensive review of the potential for incorporating mining wastes into asphalt mixtures, with a focus on steel slag, fly ash, red mud, and silica fume. The key conclusions are summarized below:

1. Sustainable Resource Utilization:
  Mining wastes, once viewed as environmentally burdensome by-products of metallurgical and mining operations, are now recognized as valuable secondary resources. Their integration into asphalt mixtures offers a promising pathway to improve pavement performance while promoting sustainable material use.
2. Environmental Benefits:
  Utilizing these wastes as alternative fillers or aggregates reduces the dependency on virgin materials, which are typically sourced through environmentally damaging quarrying activities. This contributes to lower greenhouse gas emissions, reduced habitat destruction, and less energy consumption during material extraction and processing.
3. Waste Diversion and the Circular Economy:
  Incorporating waste-derived materials into asphalt mixtures not only minimizes industrial waste stockpiling and landfilling but also supports closed-loop material recovery, aligning with circular economy principles.
4. Pollution Mitigation:
  Some mining wastes, such as red mud, pose significant environmental risks due to their high alkalinity and potential for heavy metal leaching. Repurposing these materials in asphalt pavements can prevent environmental contamination and reduce the need for costly long-term storage or remediation strategies.
5. Enhanced Material Performance:
  The inclusion of materials like steel slag and silica fume has been shown to enhance the mechanical properties of asphalt concrete, including increased stiffness, durability, and resistance to rutting and moisture damage.

In conclusion, the integration of mining waste into asphalt mixtures represents a promising path toward sustainable pavement engineering. It provides a dual advantage: enhancing the mechanical properties of road materials while addressing pressing environmental and waste management issues. The evidence presented in this study underscores the potential of materials like steel slag, fly ash, silica fume, and red mud to deliver durable, high-performance asphalt mixtures with reduced environmental footprints. As the demand for green construction continues to grow, embracing these innovative materials can help transition the asphalt industry toward a more circular, low-carbon, and resource-efficient future. With further research, supportive policies, and industry collaboration, mining waste utilization can become a mainstream practice that not only improves infrastructure resilience but also advances broader sustainability goals.

## 5. Future Research Directions

Although the mechanical, chemical, and environmental benefits of incorporating mining wastes into asphalt mixtures have been encouraging, there are a few areas that need more research to encourage broad use and optimization:

- Standardization of Characterization and Material Processing: Consistent procedures are required for the handling and classification of mining wastes, including fly ash, steel slag, and red mud. Binder compatibility and long-term performance are impacted by differences in particle size and chemical makeup. For dependable use, future research should develop uniform pre-treatment and mixing protocols.
- Long-Term Durability and Field Validation: To date, most research has been restricted to laboratory-scale assessments. Validating laboratory results requires field testing and performance monitoring in real-world settings (such as traffic, loads, and cli-mate). It is necessary to evaluate the changed mixtures’ long-term aging, rutting, and cracking behavior over prolonged service times.
- Life Cycle Assessment (LCA) and Cost–Benefit Analysis: To measure environmental savings, more thorough LCA studies spanning various waste kinds and mix designs are required. Finding the most economical and environmentally friendly waste management techniques will be made easier by combining LCA with techno-economic analysis.
- Leaching and Environmental Safety: Leaching concerns may arise from the presence of heavy metals or alkaline chemicals in some mining wastes. To guarantee safe use, long-term environmental impact assessments are necessary, particularly in applica-tions that are porous or exposed to water.
- Optimization: Studies relating to the optimal dosage of mining wastes using optimi-zation methods have not been carried out. Hence, the development of an optimal dosage is needed to ensure optimum results for road construction as well as for in-dustrial-scale production.

## Figures and Tables

**Table 1:** Chemical composition (wt%) of mining wastes used in bitumen modification [5,10,20].

Component	Red Mud	Steel Slag	Fly Ash
Iron Oxide	25–30%	15–20%	10–15%
Aluminum Oxide	10–15%	5–7%	20–25%
Silicone Oxide	8–12%	10–12%	50–60%
Calcium Oxide	2–5%	35–45%	1–3%
Sodium and Potassium Oxides	4–6%	<1%	2–4%

**Table 2:** Summary observations of chemicals used in bitumen modification.

Mining Wastes	Main Findings	References
Red Mud	High concentration of iron oxide contributes to increased binder stiffness, adhesion, and improved fatigue resistance, making it effective in enhancing the mechanical performance of asphalt mixtures.	[20]
Steel Slag	High calcium oxide is beneficial for improving moisture resistance and enhancing the alkalinity of the mixture, which gives better binder adhesion.	[10]
Fly Ash	Has the a content of aluminum oxide, which is important for pozzolanic activity and improved bonding, contributing to increased durability. Acts as strong filler material that enhances packing density and thermal stability due to high content of silicon oxide.	[5]

**Table 3:** Summary of key findings of the physical properties of modified bitumen.

Physical Property	Main Findings	References
Penetration Value	Decreased values with red mud, fly ash, and steel slag indicate increased stiffness and improved rutting resistance.	[10]
Softening Point	Increased softening point (by 8–15 °C) due to electric arc furnace slag, copper slag, and red mud improves thermal resistance.	[14,23,32]
Viscosity	Higher viscosity from fly ash/red mud improves coating, durability, and moisture resistance; warm mix and sulfur additives lower compaction temperatures.	[15,37,40]
Dynamic Shear Rheometer	Mining waste additives increase G*/sinδ and improve fatigue and rutting resistance. Increased G* and decreased δ with additives indicate improved deformation resistance.	[15,39,40,41]
Aging	Pozzolanic and antioxidant properties in red mud, fly ash, and slags mitigate oxidation and aging effects.	[42,43]

**Table 4:** XRD results of the oxide composition of red mud [5,20].

Compound	Red Mud (%)
Fe_2_O_3_	28.4
Al_2_O_3_	17.5
SiO_2_	12.6
CaO	4.8
TiO_2_	2.1
Na_2_O	4.3
K_2_O	1.9
Others	<1.0

**Table 5:** Summary of the key findings of the chemical properties of relevant mining wastes.

Properties	Key Findings	Relevant Mining Waste	References
FTIR and ATR	Detected functional groups, such as –OH, C=O, and Si–OH, indicating improved interaction between mining waste and bitumen, enhancing filler–binder bonding.	Red Mud, Steel Slag, and Silica Fume	[5,38,45]
DSC	Revealed enhanced thermal stability and altered glass transition temperatures (Tg) in modified binders, improving elasticity and temperature resistance.	Steel Slag	[12,15,46]
XRD	Identified crystalline phases like hematite, quartz, and gibbsite, confirming increased crystallinity and physical stability in modified binders.	Red Mud and Fly Ash	[10,14,44]
XRF	Quantified high levels of oxides (Fe_2_O_3_, Al_2_O_3_, SiO_2_, CaO), supporting improved stiffness, adhesion, and reduced moisture susceptibility in bitumen.	Red Mud, Fly Ash, and Steel Slag	[20,44,45]

**Table 6:** Summary of the key findings for microstructural properties.

Technique	Key Findings	References
SEM (Scanning Electron Microscopy)	Reveals rough surface morphology and particle dispersion; red mud and steel slag enhance bonding; cohesive microstructure improves durability and resistance to rutting.	[9,20,48,52]
FESEM (Field Emission Scanning Electron Microscopy)	Visualizes nanoscale features and confirms element dispersion; FESEM shows oxides, like Fe, Al, and Si, uniformly embedded, enhancing filler–matrix bonding.	[20,50,51]
MAPP (Mapping and Phase Profiling)	Combines SEM with spectroscopy to analyze phase distribution; shows uniform mineral dispersion, smoother surfaces, and fewer voids, leading to better compatibility and reduced air voids.	[12,53,54]
EDAX/EDS (Energy-Dispersive X-ray Analysis)	Identifies elemental composition, such as Ca, Si, Al, and Fe, in fillers; confirms the chemical components that enhance binder reactivity and mechanical strength.	[47]
AFM (Atomic Force Microscopy)	Analyzes surface roughness and adhesion at the nanoscale; nanostructures improve fatigue and moisture resistance; higher elasticity and stress distribution.	[49,55,56]

**Table 7:** Summary of findings of mining wastes.

Mining Waste	Key Benefits	Performance Findings	References
Steel Slag	High angularity, hardness, improves rutting resistance, Marshall stability, and moisture resistance	Used as coarse and fine aggregate; BOF and EAF slags improve fatigue life and stiffness modulus	[8,23,24,57,58]
Fly Ash	Enhances binder stiffness, improves workability and fatigue resistance, good filler replacement	Classes F and C fly ash improve water resistance; spherical particles aid mastic stiffness and packing	[10,11]
Silica Fume	Improves fatigue and rutting resistance, promotes adhesion and filler–binder interactions	Used in hot-/cold-mix; fills micro-voids, improves cohesion and aggregate adhesion	[2,12,15]
Red Mud (Iron Oxide)	Enhances thermal conductivity, aging and UV resistance, improves Marshall stability and high-temp performance	Requires additives to reduce fatigue life impact and voids; 3–7% inclusion improves rut depth and stability	[3,7,13,20,61,64,65]

**Table 8:** Summary of findings for different mining wastes.

Technique	Key Findings	References
Rutting Resistance	Steel slag improves load distribution and shear resistance; red mud enhances high-temp stiffness; 3–7% red mud reduces rut depth	[3,7,57,66]
Cracking Resistance	Silica fume and fly ash enhance binder flexibility and compaction; 6% silica fume reduces low-temp cracking	[10,11,12,28]
Fatigue Resistance	Steel slag and silica fume improve fatigue life through better binder interaction; enhances resistance to cyclic loading	[2,8,23,28]
Moisture Susceptibility	Steel slag and fly ash improve adhesion and reduce water damage; red mud needs anti-stripping agents due to porosity	[6,8,11,13,66]

## Data Availability

No new data were created or analyzed in this study. Data sharing is not applicable to this article.
